# Low-Dose Adrenaline, Promethazine, and Hydrocortisone in the Prevention of Acute Adverse Reactions to Antivenom following Snakebite: A Randomised, Double-Blind, Placebo-Controlled Trial

**DOI:** 10.1371/journal.pmed.1000435

**Published:** 2011-05-10

**Authors:** H. Asita de Silva, Arunasalam Pathmeswaran, Channa D. Ranasinha, Shaluka Jayamanne, Senarath B. Samarakoon, Ariyasena Hittharage, Ranjith Kalupahana, G. Asoka Ratnatilaka, Wimalasiri Uluwatthage, Jeffrey K. Aronson, Jane M. Armitage, David G. Lalloo, H. Janaka de Silva

**Affiliations:** 1 Clinical Trials Unit, Faculty of Medicine, University of Kelaniya, Ragama, Sri Lanka; 2 Polonnaruwa General Hospital, Polonnaruwa, Sri Lanka; 3 Kurunegala Teaching Hospital, Kurunegala, Sri Lanka; 4 Hambantota General Hospital, Hambantota, Sri Lanka; 5 Department of Primary Health Care, University of Oxford, Oxford, United Kingdom; 6 Clinical Trial Service Unit, University of Oxford, Oxford, United Kingdom; 7 Liverpool School of Tropical Medicine, Liverpool, United Kingdom; University of Melbourne, Australia

## Abstract

**Background:**

Envenoming from snakebites is most effectively treated by antivenom. However, the antivenom available in South Asian countries commonly causes acute allergic reactions, anaphylactic reactions being particularly serious. We investigated whether adrenaline, promethazine, and hydrocortisone prevent such reactions in secondary referral hospitals in Sri Lanka by conducting a randomised, double-blind placebo-controlled trial.

**Methods and Findings:**

In total, 1,007 patients were randomized, using a 2×2×2 factorial design, in a double-blind, placebo-controlled trial of adrenaline (0.25 ml of a 1∶1,000 solution subcutaneously), promethazine (25 mg intravenously), and hydrocortisone (200 mg intravenously), each alone and in all possible combinations. The interventions, or matching placebo, were given immediately before infusion of antivenom. Patients were monitored for mild, moderate, or severe adverse reactions for at least 96 h. The prespecified primary end point was the effect of the interventions on the incidence of severe reactions up to and including 48 h after antivenom administration. In total, 752 (75%) patients had acute reactions to antivenom: 9% mild, 48% moderate, and 43% severe; 89% of the reactions occurred within 1 h; and 40% of all patients were given rescue medication (adrenaline, promethazine, and hydrocortisone) during the first hour. Compared with placebo, adrenaline significantly reduced severe reactions to antivenom by 43% (95% CI 25–67) at 1 h and by 38% (95% CI 26–49) up to and including 48 h after antivenom administration; hydrocortisone and promethazine did not. Adding hydrocortisone negated the benefit of adrenaline.

**Conclusions:**

Pretreatment with low-dose adrenaline was safe and reduced the risk of acute severe reactions to snake antivenom. This may be of particular importance in countries where adverse reactions to antivenom are common, although the need to improve the quality of available antivenom cannot be overemphasized.

**Trial registration:**

www.ClinicalTrials.gov NCT00270777

*Please see later in the article for the Editors' Summary*

## Abstract 2

In a factorial randomized trial conducted in Sri Lanka, de Silva and colleagues evaluate the safety and efficacy of pretreatments intended to reduce the risk of serious reactions to antivenom following snakebite.

## Editors' Summary

**Background:**

Of the 3,000 or so snake species in the world, about 600 are venomous. Venomous snakes, which are particularly common in equatorial and tropical regions, immobilize their prey by injecting modified saliva (venom) into their prey's tissues through their fangs—specialized hollow teeth. Snakes also use their venoms for self-defense and will bite people who threaten, startle, or provoke them. A bite from a highly venomous snake such as a pit viper or cobra can cause widespread bleeding, muscle paralysis, irreversible kidney damage, and tissue destruction (necrosis) around the bite site. All these effects of snakebite are potentially fatal; necrosis can also result in amputation and permanent disability. It is hard to get accurate estimates of the number of people affected by snakebite, but there may be about 2 million envenomings (injections of venom) and 100,000 deaths every year, many of them in rural areas of South Asia, Southeast Asia, and sub-Saharan Africa.

**Why Was This Study Done?:**

The best treatment for snakebite is to give antivenom (a mixture of antibodies that neutralize the venom) as soon as possible. Unfortunately, in countries where snakebites are common (for example, Sri Lanka), antivenoms are often of dubious quality, and acute allergic reactions to them frequently occur. Although some of these reactions are mild (for example, rashes), in up to 40% of cases, anaphylaxis—a potentially fatal, whole-body allergic reaction—develops. The major symptoms of anaphylaxis—a sudden drop in blood pressure and breathing difficulties caused by swelling of the airways—can be treated with adrenaline. Injections of antihistamines (for example, promethazine) and hydrocortisone can also help. In an effort to prevent anaphylaxis, these drugs are also widely given before antivenom, but there is little evidence that such “prophylactic” treatment is effective or safe. In this randomized double-blind controlled trial (RCT), the researchers test whether low-dose adrenaline, promethazine, and/or hydrocortisone can prevent acute adverse reactions to antivenom. In an RCT, the effects of various interventions are compared to a placebo (dummy) in groups of randomly chosen patients; neither the patients nor the people caring for them know who is receiving which treatment until the trial is completed.

**What Did the Researchers Do and Find?:**

The researchers randomized 1,007 patients who had been admitted to secondary referral hospitals in Sri Lanka after snakebite to receive low-dose adrenaline, promethazine, hydrocortisone, or placebo alone and in all possible combinations immediately before treatment with antivenom. The patients were monitored for at least 96 hours for adverse reactions to the antivenom; patients who reacted badly were given adrenaline, promethazine, and hydrocortisone as “rescue medication.” Three-quarters of the patients had acute reactions—mostly moderate or severe—to the antivenom. Most of the acute reactions occurred within an hour of receiving the antivenom, and nearly half of all the patients were given rescue medication during the first hour. Compared with placebo, pretreatment with adrenaline reduced severe reactions to the antivenom by 43% at one hour and by 38% over 48 hours. By contrast, neither hydrocortisone nor promethazine given alone reduced the rate of adverse reactions to the antivenom. Moreover, adding hydrocortisone negated the beneficial effect of adrenaline.

**What Do These Findings Mean?:**

These findings show that pretreatment with low-dose adrenaline is safe and reduces the risk of acute severe reactions to snake antivenom, particularly during the first hour after infusion. They do not provide support for pretreatment with promethazine or hydrocortisone, however. Indeed, the findings suggest that the addition of hydrocortisone could negate the benefits of adrenaline, although this finding needs to be treated with caution because of the design of the trial, as does the observed increased risk of death associated with pretreatment with hydrocortisone. More generally, the high rate of acute adverse reactions to antivenom in this trial highlights the importance of improving the quality of antivenoms available in Sri Lanka and other parts of South Asia. The researchers note that the recent World Health Organization guidelines on production, control, and regulation of antivenom should help in this regard but stress that, for now, it is imperative that physicians carefully monitor patients who have been given antivenom and provide prompt treatment of acute reactions when they occur.

**Additional Information:**

Please access these Web sites via the online version of this summary at http://dx.doi.org/10.1371/journal.pmed.1000435.

- The MedlinePlus Encyclopedia has pages on snakebite and on anaphylaxis (in English and Spanish)
- The UK National Health Service Choices website also has pages on snakebite and on anaphylaxis
- The World Health Organization has information on snakebite and on snake antivenoms (in several languages); its Guidelines for the Production, Control and Regulation of Snake Antivenom Immunoglobulins are also available
- The Global Snakebite Initiative has information on snakebite
- A *PLoS Medicine* Research Article by Anuradhani Kasturiratne and colleagues provides data on the global burden of snakebite
- A *PLoS Medicine* Neglected Diseases Article by José María Gutiérrez and colleagues discusses the neglected problem of snakebite envenoming

## Introduction

Globally an estimated 421,000 envenomings and 20,000 deaths occur each year due to snakebite, although the incidence may be as high as 1,841,000 envenomings and 94,000 deaths [1]. Populations with the highest burden (in rural areas of South Asia, Southeast Asia, and sub-Saharan Africa) experience high morbidity and mortality because of poor access to often suboptimal health services; scarcity of antivenom, which is the only specific treatment for snakebite, may also be a problem [2]. The incidence of snakebite in Sri Lanka (based on hospital data) is about 200 per 100,000 individuals per year [1],[3], one of the highest in the world. In the North-Central and North-Western Provinces of the country, which have the highest incidence of bites by highly venomous snakes, three regional hospitals reported 1,851 snakebite admissions, with 11 deaths due to snakebite during 2000 [4].

Antivenom is the mainstay of treatment for snakebite. Adverse reactions to the snake antivenoms available in Sri Lanka and other countries in South Asia, which contains equine proteins, are common: both acute (anaphylactoid or pyrogenic) and delayed (serum sickness type) reactions occur [5]. Acute reactions cause the greatest problem: in most cases, symptoms are mild (urticaria, nausea, vomiting, headache, and fever), but in up to 40% of cases, severe systemic anaphylaxis may develop, including bronchospasm and hypotension [6]–[9]. In Sri Lanka, only Indian-manufactured polyvalent antivenoms are available. The rates of adverse reactions to these antivenoms are high, ranging from 43% to 81% [10]–[12]. Increasing the safety of treating individuals with snakebite using antivenom therefore has a high priority.

Prophylactic use of hydrocortisone and antihistamines before infusion of antivenom is widely practised, although the theoretical basis for this treatment is unclear and there is limited evidence of efficacy. Subcutaneous adrenaline (epinephrine) significantly reduced the incidence of acute adverse reactions in one prospective study [10], but this study was of inadequate size to establish the safety of pretreatment with adrenaline [13]. A retrospective study in Papua New Guinea suggested that adrenaline pretreatment significantly reduced acute adverse reaction rates to antivenom but that promethazine or hydrocortisone had no effect [14]. This study has subsequently been criticised for its poor design [15]. Other studies investigating the use of pretreatment with hydrocortisone or promethazine have failed to demonstrate any clear benefit [12],[16]. In view of this uncertainty about the safety and efficacy of pretreatment to reduce or prevent adverse reactions to antivenom, we conducted a large randomized, placebo-controlled, double-blind trial to determine whether low-dose adrenaline, promethazine, and hydrocortisone, alone and in all possible combinations, are significantly better than placebo in preventing acute adverse reactions to antivenom in snakebite victims.

## Methods

### Subjects and Procedures

The study was developed for secondary referral hospitals in areas in Sri Lanka with a high incidence of snakebite (Text S1). It was initiated in March 2005 at Anuradhapura, Kurunegala, and Polonnaruwa hospitals. Polonnaruwa and Kurunegala hospitals participated throughout the study to its conclusion in April 2008. The study was terminated in Anuradhapura in June 2005. Recruitment was subsequently moved to Embilipitiya hospital for the period November 2005 to May 2006, and thereafter to Hambantota hospital until the conclusion of the trial. These changes were made for a combination of administrative reasons and poor recruitment rates, and were approved at each step by the ethics review committee that approved the study. At any given time, no more than three hospitals participated in the study.

All patients who presented after snakebite were screened for eligibility by attending clinical staff (Table 1). Those over age 12 y requiring antivenom were eligible for randomisation. All participants provided written informed consent; for those unable to give consent or less than 16 y of age, a relative provided written informed consent.

**Table 1:** Inclusion and exclusion criteria.

Inclusion Criteria	Exclusion Criteria
Patients above 12 y of age	Patients who are pregnant or nursing
Patients admitted to hospital after snakebite in whom antivenom is indicated	Patients who are currently taking beta- or alpha-adrenoceptor antagonists, or tricyclic antidepressants
Patients who give informed consent	Patients in whom adrenaline may be contraindicated (this may include patients with the following): (1) history of ischaemic heart disease or stroke, (2) uncontrolled hypertension, (3) tachyarrhythmias

The primary aim was to determine whether low-dose adrenaline (0.25 ml of a 1∶1,000 solution subcutaneously; i.e., 250 micrograms), promethazine (25 mg intravenously), or hydrocortisone (200 mg intravenously), alone or in combination, given as pretreatment, significantly reduced severe adverse reactions to antivenom compared with placebo (0.9% NaCl) up to and including 48 h. All time points relate to time after starting the antivenom infusion. Adverse reactions to antivenom were predefined as mild, moderate, and severe based on an international classification of anaphylaxis reactions [17] (Table 2). We also assessed the safety of the pretreatment medication, looking specifically for complications that might be caused by adrenaline: arrhythmias, increased systolic blood pressure (BP) (>30 mm Hg increase), and intracerebral haemorrhage.

**Table 2:** Classification of acute adverse reactions to antivenom.

Mild	Moderate	Severe
Facial oedema	Abdominal pain	Drowsiness or altered consciousness
Pruritus	Nausea	Systolic BP < 80 mm Hg
Urticaria	Vomiting	Cyanosis
Fever^a^	Bronchospasm	Confusion
Rigor^a^	Stridor	

a Not in original classification [17] but added to capture all of the systemic reactions.

**Figure 1:**
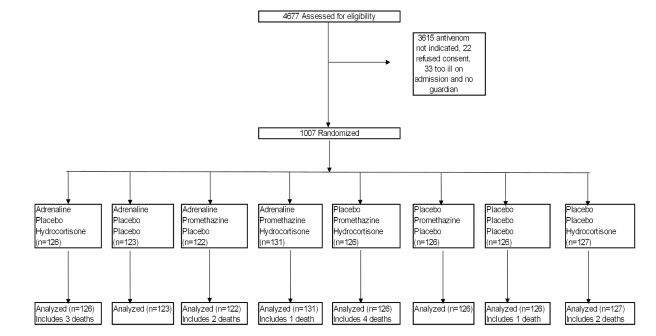
Trial profile.

Patients were randomized with equal probability to one of eight different treatments in a 2×2×2 factorial blinded design, using a triple-dummy technique (Figure 1). Stratified block randomization was done by hospital site. For each site, computer-generated random allocation sequences were prepared independently by the trial statistician. All trial medications were prepared at the Clinical Trials Unit, Faculty of Medicine, University of Kelaniya, and packaged in identical sealed envelopes. Syringes containing adrenaline and adrenaline placebo were clearly marked to ensure that they were not administered intravenously. The envelopes, with unique, centre-specific identification numbers, were stored on site.

Patients were seen by ward doctors within 10 min of admission and examined. Baseline investigations, such as electrocardiography and assessment of blood clotting, were done as indicated. Randomization occurred after clinical assessment by ward doctors and after written informed consent had been obtained. Patients remained under the care of consultant physicians following management protocols based on current treatment guidelines that had been approved by the study team. The ward team made all clinical decisions relating to patient care and administered the pretreatment medication and antivenom. Monitoring for acute reactions was carried out independently by groups of three medically qualified clinical research coordinators dedicated to each site who were blind to the interventions. Participants were observed continuously for the first 2 h and then reviewed at 4-h intervals until 48 h.

Patients were given ten vials of antivenom dissolved in 500 ml of isotonic saline as an intravenous infusion over 1 h. Antivenom treatment was repeated as deemed necessary by attending clinicians, according to clinical judgement. However, patients were not given further doses of trial medication, even if antivenom was repeated. Patients were monitored using a clinical observation protocol developed jointly by consultant physicians and study coordinators for acute adverse reactions to antivenom and any adverse reactions to the study drugs. Study-related patient information was recorded in standardised clinical record forms.

Patients were kept in hospital for at least 96 h after the infusion of antivenom. If a reaction developed during infusion, or if a patient developed cardiac arrhythmias, ischaemic changes on the electrocardiogram, a rise in BP (for systolic, an increase of >30 mm Hg, or for diastolic, an increase of >20 mm Hg, from pretreatment level), a fall in BP (for systolic, a decrease of >20 mm Hg, or for diastolic, a decrease of >10 mm Hg, from pretreatment level), or anaphylaxis after the study drug and antivenom, appropriate treatment (“rescue medication”) was given solely at the discretion of the attending clinicians. Reactions to antivenom were treated by stopping the antivenom infusion temporarily, and giving, alone or in any combination, 0.25 ml (mild reactions) or 0.5 ml (moderate and severe reactions) of 1∶1,000 adrenaline intramuscularly, 25 mg of promethazine intravenously, or 200 mg of hydrocortisone intravenously (rescue medication).

Ethics committee approval was received from the Ethics Review Committee, Faculty of Medicine, University of Kelaniya. An independent data monitoring committee was provided with interim analyses when information from groups of 200 new patients became available. In the light of these analyses and the results of any other new relevant information, the data monitoring committee was instructed to advise the principal investigator if, in the committee's view, there was proof beyond reasonable doubt that the data showed that any part of the protocol under investigation became clearly indicated or contraindicated, either for all participants or for a specific subgroup of trial participants, or if it appeared that no clear outcome would be obtained. However, no data monitoring committee–driven changes to protocol were made as a result of interim analyses.

### Statistical Analysis

#### Sample size calculations

We estimated that acute adverse reactions would occur in about 40% of patients who received antivenom and that a reduction of over 25% in the rate of acute adverse reactions would correspond to a substantial benefit. Using the proposed design, a sample size of 1,000 gave 80% power to detect a 25% relative reduction in adverse reactions from the current reaction rate by any one treatment, at *p<*0.01.

#### Analysis

The prespecified primary outcome measure was the frequency of severe reactions to antivenom up to and including 48 h after antivenom administration in those allocated to each treatment compared to those not allocated to that treatment. Secondary outcomes were rates of severe reactions within 1 or 6 h, rates of any adverse reactions up to and including 48 h, and acute adverse reactions to study treatments separately (prespecified as arrhythmias, intracerebral haemorrhage, or an increase in systolic BP>30 mm Hg). The 2×2×2 factorial design used for this trial facilitates primary analyses to determine the main effects of the three treatments, and allows investigation of two-way and three-way interactions.

Analyses were undertaken on an intention-to-treat basis using logistic regression, and took into account clustering by trial site. The final model included the three trial medications and all three two-way interaction terms. Odds ratios (ORs) and 95% confidence intervals for the effects of each treatment and the two-way interactions were calculated. This superseded our original intention to compare event rates for those who received a particular drug versus those not given that drug, and to repeat these analyses with stratification by other treatments administered to check for interactions between trial medications. This change was made on the advice given by the statistical reviewer for the journal.

No allowance was to be made for multiple comparisons in the primary analyses but for secondary and, particularly, for tertiary comparisons, allowance was made for multiple hypothesis testing, taking into account the nature of the events (including timing, duration, and severity) and evidence from other studies.

## Results

From March 2005 to April 2008, 4,677 patients who presented after snakebite to trial hospitals were screened, and 1,007 eligible patients were randomized (53 at Anuradhapura, 16 at Embilipitiya, 152 at Hambantota, 353 at Kurunegala, and 433 at Polonnaruwa) (Text S2). The main reason for exclusion was lack of clinical indication for antivenom. Recruitment was stopped when the target sample size of 1,000 was reached in April 2008. All the randomized patients completed the study and were evaluated; there were no protocol deviations.

Table 3 shows the baseline demographic and other clinical characteristics in the three treatment groups and shows good balance between the groups. The median time from snakebite to administration of antivenom was similar at the different hospitals (median time ranged from 3.9 to 4.6 h). More than 70% of patients were transferred from smaller rural hospitals. Some of them had received antivenom (20% of all study patients), hydrocortisone (25% of all study patients), or promethazine (9.7% of all study patients) before transfer to a trial hospital. None of the patients had been given adrenaline. This did not have a significant effect on the trial outcomes. The biting snake species was identified in only 25% of the cases.

**Table 3:** Patient baseline characteristics by treatment allocation.

Characteristic	Adrenaline		Hydrocortisone		Promethazine		Total (*n = *1,007)
	Yes (*n = *502)	No (*n = *505)	Yes (*n = *510)	No (*n = *497)	Yes (*n = *505)	No (*n = *502)	
Male, *n* (%)	392 (78.1)	384 (76.0)	388 (76.1)	388 (78.1)	383 (75.8)	393 (78.3)	776 (77.1)
Age in years, mean (standard deviation)	36.0 (13.6)	37.1 (13.5)	36.0 (13.4)	37.1 (13.7)	36.8 (13.8)	36.3 (13.4)	36.5 (13.6)
Time between bite and antivenom in hours, median (interquartile range)	4.3 (2.8–6.8)	4.3 (2.9–6.8)	4.3 (2.8–6.7)	4.3 (3.0–7.2)	4.2 (2.8–7.9)	4.4 (3–6.9)	4.3 (2.9–6.8)
Direct admission, *n* (%)	136 (27.1)	134 (26.5)	141 (27.7)	129 (26.0)	155 (30.7)	115 (22.9)	270 (26.8)
History of previous snakebite, *n* (%)	51 (10.2)	54 (10.7)	54 (10.6)	51 (10.3)	60 (11.8)	45 (9.0)	105 (10.4)
Snake identified (%)	135 (26.9)	124 (24.6)	126 (24.7)	133 (26.7)	124 (24.6)	135 (26.9)	259 (25.7)
Antivenom given before transfer, *n* (%)	102 (20.3)	103 (20.4)	96 (18.8)	109 (21.9)	95 (18.8)	110 (21.9)	205 (20.4)
Hydrocortisone given before transfer, *n* (%)	127 (25.3)	128 (25.4)	131 (25.7)	124 (25.0)	117 (23.2)	138 (27.5)	255 (25.3)
Promethazine given before transfer, *n* (%)	47 (9.4)	51 (10.1)	45 (8.8)	53 (10.7)	46 (9.1)	52 (10.3)	98 (9.7)
History of allergy, *n* (%)	37 (7.4)	45 (8.9)	39 (7.7)	43 (8.7)	42 (8.3)	40 (8.0)	82 (8.1)
History of bronchial asthma, *n* (%)	25 (5.0)	32 (6.3)	32 (6.3)	25 (5.0)	30 (5.9)	27 (5.4)	57 (5.7)

**Figure 2:**
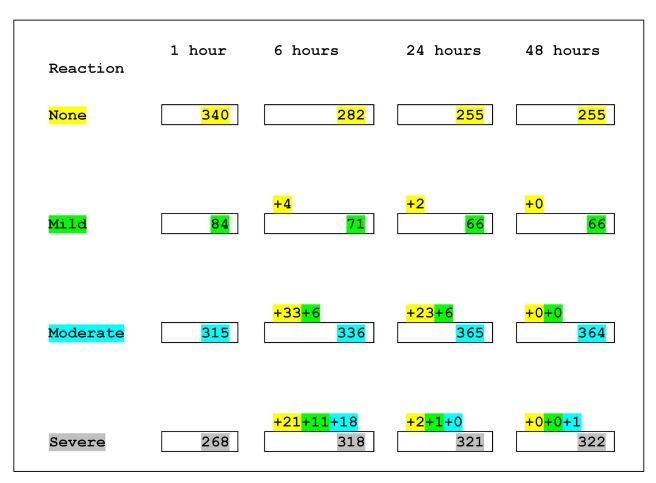
Progression of type of reaction over 48 h. Numbers within boxes indicate the number of patients according to the highest category of reaction they had experienced by that time. Numbers above the boxes indicate the number of patients who experienced a higher category of reaction during the preceding interval. Those who changed from no reaction to a reaction category are indicated by numbers highlighted in yellow. Those who changed from mild reaction to a higher category are indicated by numbers highlighted in green. Those who changed from moderate to severe reaction are indicated by numbers highlighted in turquoise. For example the above numbers can be interpreted as follows. At 1 h, there were 315 patients with moderate reaction, and by 6 h, 336 patients were classified as moderate reactions; 33 patients who had had no reaction in 1 h had a moderate reaction during this interval, six patients who had had mild reaction in 1 h had a moderate reaction during this interval, and 18 patients who had had moderate reaction in 1 h had a severe reaction during this interval: 336 = (315+33+6)−18.

In total, 752 patients (75%) developed acute reactions to antivenom within 48 h of administration (Table 4), of which 667 reactions (almost 90%) occurred in the first hour (Figure 2). Of these, 9% were mild reactions, 48%, moderate, and 43%, severe; 83% of severe reactions occurred in the first hour. After the first hour the category of reaction changed in 128 patients (12.7%); this change in reaction category took place before the end of 6 h in 93 of these 128 patients (Figure 2). There was a change in reaction category after 6 h in only 35 patients, and this included the one patient whose reaction category changed from moderate to severe during the second 24 h of observation. Patients were given rescue medication at the discretion of the attending clinicians and managed as clinically indicated. In all, 40% of patients received rescue medication within the first hour: 27% of all patients with mild or no acute reactions, 47% of all patients with moderate reactions, and 50% of all patients with severe reactions.

**Table 4:** Outcomes during first hour and 48 h by treatment allocation.

Outcome	Reaction	Adrenaline		Hydrocortisone		Promethazine		Total (*n = *1,007)
		Yes (*n = *502)	No (*n = *505)	Yes (*n = *510)	No (*n = *497)	Yes (*n = *505)	No (*n = *502)	
Reaction during first hour	None, *n* (%)	185 (36.9)	155 (30.7)	170 (33.3)	170 (34.2)	182 (36.0)	158 (31.5)	340 (33.8)
	Mild, *n* (%)	43 (8.6)	41 (8.1)	39 (7.7)	45 (9.1)	29 (5.7)	55 (11.0)	84 (8.3)
	Moderate, *n* (%)	154 (30.7)	161 (31.9)	164 (32.2)	151 (30.4)	167 (33.1)	148 (29.5)	315 (31.3)
	Severe, *n* (%)	120 (23.9)	148 (29.3)	137 (26.9)	131 (26.4)	127 (25.2)	141 (28.1)	268 (26.6)
	Any reaction, *n* (%)	317 (63.1)	350 (69.3)	340 (66.7)	327 (65.8)	323 (64.0)	344 (68.5)	667 (66.2)
	OR^a^ (95% CI) for severe reaction	0.57 (0.43–0.75)		0.86 (0.60–1.24)		0.81 (0.51–1.30)		
	OR^a^ (95% CI) for any reaction	0.76 (0.64–0.91)		1.04 (0.85–1.28)		0.81 (0.65–1.02)		
Reaction during 48 h	None, *n* (%)	135 (26.9)	120 (23.7)	126 (24.7)	129 (26.0)	128 (25.4)	127 (25.3)	255 (25.3)
	Mild, *n* (%)	29 (5.8)	37 (7.3)	30 (5.9)	36 (7.2)	22 (4.4)	44 (8.8)	66 (6.6)
	Moderate, *n* (%)	184 (36.7)	180 (35.6)	188 (36.9)	176 (35.4)	197 (39.0)	167 (33.3)	364 (36.2)
	Severe, *n* (%)	154 (30.7)	168 (33.3)	166 (32.5)	156 (31.4)	158 (31.3)	164 (32.7)	322 (32.0)
	Any reaction, *n* (%)	367 (73.1)	385 (76.3)	384 (75.3)	368 (74.0)	377 (74.6)	375 (74.7)	752 (74.7)
	OR^a^ (95% CI) for severe reaction	0.62 (0.51–0.74)		0.80 (0.53–1.21)		0.87 (0.50–1.52)		
	OR^a^ (95% CI) for any reaction	0.85 (0.71–1.00)		1.07 (0.87–1.32)		1.00 (0.74–1.35)		
Rescue medication during first hour		206 (41.0)	191 (37.8)	186 (36.5)	211 (42.5)	194 (38.4)	203 (40.4)	
		*X* ^2^ = 1.09; *p = *0.30		*X* ^2^ = 3.77; *p* = 0.052		X^2^ = 0.43; *p = *0.51		
Time (min) to rescue medication, mean (standard error)		30.7 (2.2)	25.9 (1.6)	31.1 (2.1)	25.4 (1.7)	30.7 (2.1)	25.8 (1.7)	
		*t* = 1.78; *p* = 0.08		*t* = 2.13; *p* = 0.03		*t* = 1.86; *p* = 0.06		

All time points relate to time after starting the antivenom infusion.

a For predictors of severe reaction, ORs were calculated using the main effects and all two-way interactions of the trial medications; for predictors of any reaction, ORs were calculated using only the main effects of the trial medications because there were no significant interactions.

Adrenaline reduced the rate of severe adverse reactions compared with placebo at 1 h by 43% (OR 0.57, 95% CI 0.43–0.75; *p<*0.001); and by 38% over 48 h (OR 0.62, 0.51–0.74; *p<*0.001) (Tables 5 and 6). Neither hydrocortisone nor promethazine had any significant effect on the risk of severe adverse reactions at 1 h or 48 h (Tables 5 and 6). The same pattern was observed at 6 and 24 h (Tables S1 and S2, respectively). There was some evidence that the effect of allocation to hydrocortisone in addition to adrenaline negated the benefit of adrenaline (OR 1.50, 95% CI 1.09–2.07; *p = *0.013). Furthermore, adrenaline, but neither hydrocortisone nor promethazine, reduced the rate of all reactions, especially at 1 h (Table 4).

**Table 5:** Risk of severe reaction during the first hour by treatment: main effects and two-way interactions adjusted for clustering by trial site.

Treatment	Severe Reaction			Logistic Regression Model, Main Effects and Two-Way Interactions		
	Yes	No	Total	OR	95% CI	*p*-Value
Adrenaline	28	95	123	0.57	0.43–0.75	<0.001
Hydrocortisone	39	88	127	0.86	0.60–1.24	0.430
Promethazine	37	89	126	0.81	0.51–1.30	0.378
Adrenaline and hydrocortisone	33	93	126	1.50	1.09–2.07	0.013
Adrenaline and promethazine	25	97	122	1.17	0.85–1.61	0.327
Hydrocortisone and promethazine	31	95	126	0.97	0.64–1.47	0.896
Adrenaline, hydrocortisone, and promethazine	34	97	131			
Triple placebo	41	85	126			
Total	268	739	1,007			

There were no three-way interactions. Data are from five hospitals. All time points relate to time after starting the antivenom infusion.

**Table 6:** Risk of severe reaction up to and including 48 h by treatment: main effects and two-way interactions adjusted for clustering by trial site.

Treatment	Severe Reaction			Logistic Regression Model, Main Effects and Two-Way Interactions		
	Yes	No	Total	OR	95% CI	*p*-Value
Adrenaline	33	90	123	0.62	0.51–0.74	<0.001
Hydrocortisone	41	86	127	0.80	0.53–1.21	0.296
Promethazine	43	83	126	0.87	0.50–1.52	0.629
Adrenaline and hydrocortisone	43	83	126	1.76	1.24–2.50	0.002
Adrenaline and promethazine	33	89	122	1.16	0.80–1.69	0.441
Hydrocortisone and promethazine	37	89	126	1.00	0.65–1.55	0.999
Adrenaline, hydrocortisone, and promethazine	45	86	131			
Triple placebo	47	79	126			
Total	322	685	1,007			

There were no three-way interactions. Data are from five hospitals. All time points relate to time after starting the antivenom infusion.

Adrenaline and promethazine seemed to be safe (Table 7): only 13 (1.3%) patients died. All deaths were considered by the supervising physician to be consequences of envenoming or complications that developed during intensive care treatment for envenoming (one death from pneumonia, four from sepsis, three from shock, three from acute renal failure, and two from respiratory failure). There were significantly more deaths among those who received hydrocortisone compared to no hydrocortisone (ten [2%] versus three [0.6%]; OR 3.3, 95%CI 1.28–8.52; *p* = 0.014) (Table 8). In all, 261 patients had a significant rise in BP (increase in systolic BP of >30 mm Hg and/or increase in diastolic BP of >20 mm Hg) within 48 h, but there was no significant association between rise in BP and trial medications, individually or combined at 30 or 60 min (Table 7). No patient had an intracerebral haemorrhage or arrhythmia. There was no significant difference in the use of rescue medication between the treatment groups.

**Table 7:** Heart rate, blood pressure, and number of patients with rise in blood pressure at 30 min and 60 min after pretreatment administered.

Time after Pretreatment	Measure	Adrenaline		Hydrocortisone		Promethazine	
		Yes (*n = *502)	No (*n = *505)	Yes (*n = *505)	No (*n = *502)	Yes (*n = *505)	No (*n = *502)
30 min	Heart rate	94.9 (0.91)	94.9 (0.93)	96.2 (0.95)	93.7 (0.89)	95.2 (0.94)	94.6 (0.90)
	Systolic BP	114.9 (0.97)	111.5 (1.01)	113.9 (0.98)	112.6 (1.00)	113.5 (1.02)	112.9 (0.95)
	Diastolic BP	70.3 (0.68)	68.9 (0.66)	70.3 (0.68)	69.0 (0.65)	69.4 (0.67)	69.9 (0.67)
	Number of patients with rise in BP^a^	63 (12.6)	52 (10.3)	63 (12.4)	52 (10.4)	66 (13.1)	49 (9.8)
60 min	Heart rate	93.4 (0.85)	93.0 (0.88)	94.2 (0.88)	92.3 (0.85)	93.4 (0.85)	93.1 (0.87)
	Systolic BP	117.1 (0.85)	114.2 (0.94)	115.6 (0.93)	115.6 (0.87)	116.5 (0.91)	114.7 (0.89)
	Diastolic BP	71.5 (0.61)	69.8 (0.64)	71.2 (0.65)	70.2 (0.61)	70.8 (0.63)	70.6 (0.63)
	Number of patients with rise in BP^a^	84 (16.7)	64 (12.7)	82 (16.1)	66 (13.3)	85 (16.8)	63 (12.6)

All values are mean (standard error).

a An increase in systolic BP of >30 mm Hg and/or diastolic BP of >20 mm Hg higher than baseline.

**Table 8:** Risk of death by treatment: main effects adjusted for clustering by trial site.

Treatment	Death			Logistic Regression Model, Main Effects		
	Yes	No	Total	OR	95% CI	*p*-Value
Adrenaline	0	123	123	0.85	0.39–1.85	0.681
Hydrocortisone	2	125	127	3.30	1.28–8.52	0.014
Promethazine	0	126	126	1.16	0.80–1.68	0.220
Adrenaline and hydrocortisone	3	123	126			
Adrenaline and promethazine	2	120	122			
Hydrocortisone and promethazine	4	122	126			
Adrenaline, hydrocortisone, and promethazine	1	130	131			
Triple placebo	1	125	126			
Total	13	994	1,007			

Data from five hospitals.

## Discussion

Reactions to antivenom present considerable challenges to clinicians treating snakebite. The frequency of early reactions varies markedly between individual antivenoms and between different batches of antivenom from the same manufacturer, occurring with a frequency that ranges from less than 0.5% up to 87%, although only a small proportion of reactions are life threatening [7]. The high reaction rates of 75% observed in this study are in line with the rates of between 43% and 81% that were observed in three previous Sri Lankan studies [10]–[12].

Given such high rates of antivenom reactions in some settings, it is not surprising that pharmacological prophylaxis has been advocated to reduce acute adverse reactions to antivenom. Before this study, only the routine use of adrenaline was supported by any evidence. Low-dose subcutaneous adrenaline given immediately before antivenom to snakebite victims significantly reduced the incidence of acute adverse reactions to the antivenom from 43% to 11% [10]. However, the study included only 102 participants, primarily observed for the first hour after infusion, and could not establish safety, a major concern regarding the use of adrenaline as a prophylactic agent [13], particularly the risk of intracerebral haemorrhage [18],[19]. Although a recent study from Papua New Guinea suggested that adrenaline pretreatment was effective [14], the retrospective design, lack of standardised definitions, and a selective statistical analysis that did not correct for multiple comparisons make it difficult to draw firm conclusions from this study.

Prophylactic use of hydrocortisone and antihistamines before infusion of antivenom is widely implemented. However, one small randomized controlled trial demonstrated no benefit from the routine use of antihistamines [16]. Hydrocortisone takes several hours to act and may be ineffective as a prophylactic against acute adverse reactions that develop almost immediately after antivenom treatment. One small study (52 patients) showed that intravenous hydrocortisone alone was ineffective in preventing acute adverse reactions to antivenom, but demonstrated a trend towards hydrocortisone reducing reactions when given with intravenous chlorphenamine [12]. However, all of the reactions were mild or moderate, and the trial was not designed to study the efficacy of chlorphenamine alone, making it difficult to interpret the results.

In contrast to these small studies, our trial enrolled just over 1,000 patients, and 752 patients experienced reactions. Our prespecified primary end point was the development of severe reactions to antivenom during the first 48 h after its administration. However, our data clearly showed that more than 80% of severe reactions occurred during the first hour after antivenom administration, and only a negligible number of severe reactions occurred more than 6 h after antivenom administration. Furthermore, about 40% of patients were given rescue medication (i.e., adrenaline, hydrocortisone, or promethazine as rescue medication irrespective of the randomization) in the first hour after antivenom administration. Such early administration of rescue medication may have diluted the effects of the randomization on reactions at the later time points, but should not have affected rates of reactions at 1 h, and we therefore chose to focus on severe reactions during the first hour. Previous studies have used a variety of different definitions for reactions, and we chose to use an established international grading [17] in an attempt to standardise this; rates of severity of reactions are therefore not directly comparable to previous studies. The factorial design enabled us to investigate both direct effects and interactions between the different medications in the most efficient manner.

We found that administration of adrenaline significantly and substantially reduced the risk of severe adverse reactions in the first hour and that this was still apparent at 48 h, but neither hydrocortisone nor promethazine had any clear effect. We have also unequivocally demonstrated that a dose of subcutaneous adrenaline of 250 micrograms is safe after snakebite, even where there is coagulopathy. While pretreatment with hydrocortisone or promethazine did not reduce severe reaction rates to antivenom significantly, hydrocortisone negated the beneficial effects of adrenaline when these treatments were given together. However, given the multiple comparisons and post-hoc nature of this finding, it should be interpreted cautiously. Hydrocortisone was also associated with an increased risk of death, but this finding was based on very small numbers. Given that hydrocortisone has no benefit and may even be harmful, we would discourage its current widespread empirical use as a pretreatment before antivenom administration.

The mechanism of reactions to antivenom is uncertain. Acute reactions may be due to type 1 (IgE-mediated) hypersensitivity, but antivenom reactions often occur in those with no previous exposure to equine proteins. Although some commercial antivenoms have anticomplement activity in vitro, complement activation has never been clearly demonstrated in patients with antivenom reactions [6],[20],[21]. Early reactions are most likely to be due to a combination of type 1 hypersensitivity, complement activation, and the effect of aggregates of immunoglobulin or immunoglobulin fragments, including Fc, which can be found in even highly refined antivenoms [22]. Although theoretically cleaving of the IgG molecule into smaller fragments should reduce the incidence of antivenom reactions, this has not been shown in clinical studies, and the major influence on reaction rates appears to be the manufacturing process [7]: there is emerging evidence that the use of caprylic acid, which results in a more pure IgG preparation, may reduce reaction rates [23],[24]. Slow infusion of antivenom intravenously (rather than administration by bolus injection) has also been advocated as a way of reducing reaction rates, although the only small comparative study of methods of administration found no difference in the rates and severity of reactions between a 30-min infusion and intravenous injection over 10 min. Using a small test dose of antivenom to detect patients who may develop acute adverse reactions to the antivenom has no predictive value and can itself cause anaphylactic reactions [6],[25].

The high rate of adverse reactions to antivenom observed in our study is common to large areas of South Asia, and is an example of how poor manufacturing and quality control by antivenom producers causes substantial problems for patients and their doctors. This highlights the importance of addressing issues of poor quality and potentially unsafe antivenom. Even well-manufactured antivenom may be associated with severe adverse reaction rates of up to 5% [15]. We therefore welcome the recent World Health Organization guidelines on production, control, and regulation of antivenom [26]. The need for concerted action by local health and regulatory authorities, the World Health Organization, and other stakeholders, including technology transfer programmes between antivenom manufacturers, to improve the quality of antivenom can not be overemphasized. Ultimately, the prevention of antivenom reactions will depend on improving the quality of antivenom. The increasing recognition of the considerable burden of snakebite and its treatment will hopefully lead to such improvements. Until these overdue improvements come about, we have shown that pretreatment with low-dose adrenaline is an effective and safe therapy to prevent acute reactions to antivenom. This finding may be of particular relevance in areas where adverse reactions to antivenom are common. Meanwhile, we continue to reiterate that the need for careful observation of patients receiving antivenom and prompt treatment of acute reactions when they occur remains undiminished.

## Supporting Information

Table S1 Risk of severe reaction during the first 6 h by treatment. Main effects and two-way interactions adjusted for clustering by trial site.
(0.04 MB DOC)

Table S2 Risk of severe reaction during first 24 h by treatment. Main effects and two-way interactions adjusted for clustering by trial site.
(0.04 MB DOC)

Text S1 Study protocol.
(0.10 MB DOC)

Text S2 CONSORT checklist.
(0.22 MB DOC)

## Abbreviations

BP: blood pressure
OR: odds ratio
