# Supplementary material for: Low-Dose Adrenaline, Promethazine, and Hydrocortisone in the Prevention of Acute Adverse Reactions to Antivenom following Snakebite: A Randomised, Double-Blind, Placebo-Controlled Trial
Source: PLoS Med. 2011 May 10;8(5):e1000435. doi: 10.1371/journal.pmed.1000435 (PMC3091849; doi:10.1371/journal.pmed.1000435)
Supplement: Table S1 — Risk of severe reaction during the first 6 h by treatment. Main effects and two-way interactions adjusted for clustering by trial site. (0.04 MB DOC) [file pmed.1000435.s001.doc]

Table S1. Risk of severe reaction during the first 6 hours by treatment – main effects and two way interactions* adjusted for clustering by trial site#

|  | Severe reaction | | |  | Logistic regression model, main effects and 2 way interactions | | |
| --- | --- | --- | --- | --- | --- | --- | --- |
|  | Yes | No | Total |  | OR | 95% CI | p-value |
| Adrenaline | 32 | 81 | 123 |  | 0.59 | 0.50 – 0.70 | <0.001 |
| Hydrocortisone | 41 | 86 | 127 |  | 0.80 | 0.53 – 1.21 | 0.296 |
| Promethazine | 43 | 83 | 126 |  | 0.87 | 0.50 – 1.52 | 0.627 |
| Adrenaline & Hydrocortisone | 43 | 83 | 126 |  | 1.84 | 1.34 – 2.52 | <0.001 |
| Adrenaline & Promethazine | 33 | 89 | 122 |  | 1.21 | 0.81 – 1.81 | 0.351 |
| Hydrocortisone & Promethazine | 35 | 91 | 126 |  | 0.93 | 0.58 – 1.48 | 0.748 |
| Adrenaline, Hydrocortisone & Promethazine | 44 | 87 | 131 |  |  |  |  |
| Triple placebo | 47 | 79 | 126 |  |  |  |  |
| Total | 318 | 689 | 1007 |  |  |  |  |

* There was no three-way interactions

# Five hospitals
