# Supplementary material for: Low-Dose Adrenaline, Promethazine, and Hydrocortisone in the Prevention of Acute Adverse Reactions to Antivenom following Snakebite: A Randomised, Double-Blind, Placebo-Controlled Trial
Source: PLoS Med. 2011 May 10;8(5):e1000435. doi: 10.1371/journal.pmed.1000435 (PMC3091849; doi:10.1371/journal.pmed.1000435)
Supplement: Table S2 — Risk of severe reaction during first 24 h by treatment. Main effects and two-way interactions adjusted for clustering by trial site. (0.04 MB DOC) [file pmed.1000435.s002.doc]

Table S2. Risk of severe reaction during first 24 hours by treatment – main effects and two way interactions* adjusted for clustering by trial site#

|  | Severe reaction | | |  | Logistic regression model, main effects and 2 way interactions | | |
| --- | --- | --- | --- | --- | --- | --- | --- |
|  | Yes | No | Total |  | OR | 95% CI | p-value |
| Adrenaline | 33 | 90 | 123 |  | 0.62 | 0.52 – 0.74 | <0.001 |
| Hydrocortisone | 41 | 86 | 127 |  | 0.81 | 0.53 – 1.22 | 0.314 |
| Promethazine | 43 | 83 | 126 |  | 0.88 | 0.50 – 1.55 | 0.654 |
| Adrenaline & Hydrocortisone | 43 | 83 | 126 |  | 1.73 | 1.23 – 2.44 | 0.002 |
| Adrenaline & Promethazine | 33 | 89 | 122 |  | 1.14 | 0.79 – 1.64 | 0.485 |
| Hydrocortisone & Promethazine | 37 | 89 | 126 |  | 0.98 | 0.62 – 1.57 | 0.945 |
| Adrenaline, Hydrocortisone & Promethazine | 44 | 87 | 131 |  |  |  |  |
| Triple placebo | 47 | 79 | 126 |  |  |  |  |
| Total | 321 | 686 | 1007 |  |  |  |  |

* There was no three-way interactions

# Five hospitals
