# Supplementary material for: Low-Dose Adrenaline, Promethazine, and Hydrocortisone in the Prevention of Acute Adverse Reactions to Antivenom following Snakebite: A Randomised, Double-Blind, Placebo-Controlled Trial
Source: PLoS Med. 2011 May 10;8(5):e1000435. doi: 10.1371/journal.pmed.1000435 (PMC3091849; doi:10.1371/journal.pmed.1000435)
Supplement: Text S1 — Study protocol. (0.10 MB DOC) [file pmed.1000435.s003.doc]

**Low dose adrenaline, promethazine, & hydrocortisone (alone and in combination) to prevent acute adverse reactions to antivenom in people bitten by snakes: randomised, double blind, placebo-controlled trial**

**Trial Steering Committee (TSC):**

**H A de Silva**

**J K Aronson**

**J Armitage**

**C D Ranasinha**

**D G Lalloo**

**H J de Silva (Chairman)**

**Data Monitoring Committee (DMC):**

**C Warlow (Chairman)**

**S B Gunatilake**

**R Wickremasinghe (Statistician)**

**Trial Statistician:**

**A Pathmeswaran**

**Participating institutions:**

**Faculty of Medicine, University of Kelaniya, Sri Lanka**

**Polonnaruwa General Hospital, Sri Lanka**

**Kurunegala General Hospital, Sri Lanka**

**Hambantota Base Hospital, Sri Lanka**

# Liverpool School of Tropical Medicine, Liverpool, UK

# Department of Primary Health Care & the Clinical Trial Service Unit, University of Oxford, UK

**Expected start date : February 2005**

**Expected trial duration : 30-36 months**

**SYNOPSIS**

| **Title** | **Low dose adrenaline, promethazine, & hydrocortisone (alone and in combination) to prevent acute adverse reactions to antivenom in people bitten by snakes: randomised, double-blind, placebo-controlled trial** |
| --- | --- |
| Aim | Investigation of the efficacy and safety of pretreatment with adrenaline, promethazine & hydrocortisone (alone and in combination) in comparison with placebo to prevent acute adverse reactions to antivenom |
| Design | Type: a randomised, double-blind, placebo-controlled study  Number of centres: three  Estimated duration: six months – one year  Design: factorial 2x2x2 design |
| Sample size | At least 1000 patients are required to have 80% power at p<0.01 to detect a 25% reduction in the rate of acute adverse reactions to antivenom |
| Indication | Snakebite with evidence of systemic / severe local envenomation |
| Patient selection I | Inclusion criteria   - Age above 12 yrs - Patients admitted to hospital after snakebite in whom antivenom is indicated - Patients who give informed consent |
| Patient selection II | Exclusion criteria   - Patients in whom adrenaline may be contraindicated - Patients currently taking beta- or alpha-adrenoceptor antagonists, and tricyclic antidepressants - Pregnant and nursing women |
| Trial medication | Drugs: adrenaline, promethazine, & hydrocortisone alone and in combination  Placebo: 0.9% NaCl  Duration of treatment: once as pretreatment before antivenom |
| Efficacy variables | Efficacy:   - Reduction in acute adverse reactions to antivenom   Safety:   - Adverse reactions to trial medications |

**BACKGROUND TO THE STUDY**

The overall incidence of snakebite in Sri Lanka exceeds 400 per 100,000 population per year, one of the highest in the world. In the North-Central and North-Western provinces of the country, where snakebite incidence is particularly high, three regional hospitals reported 1851 admissions with 11 deaths due to snakebite during 2000 [Ministry of Health, Sri Lanka, 2002]. Although there are more than 90 species of snakes in the country, much of the morbidity and about 95% of the mortality associated with snakebites are due to the highly venomous cobra, Russell’s viper, and kraits [De Silva & Ranasinghe, 1983].

Polyvalent antivenom is the most effective, if not the only effective treatment available for snakebite envenomation in Sri Lanka. Acute adverse reactions to the antivenom are common [Karunaratne & Anandadas, 1973] and include anaphylaxis. Reaction rates ranging from 43% [Premawardena et al., 1999] to 81% [Ariaratnam et al., 2001] have been reported. In most cases symptoms are mild: urticaria, nausea, vomiting, diarrhoea, headache, and fever; but in up to 40% of cases severe systemic anaphylaxis develops, with bronchospasm, hypotension, or angio-oedema [Malasit et al., 1986]. Even though Fab antivenoms are thought to be less immunogenic, a recent comparative study reported an acute reaction rate of 48% [Ariaratnam et al., 2001]. As a result, the management of acute adverse reactions to antivenom is an important part of the management of snake envenoming in Sri Lanka. Increasing the safety of treatment with antivenom for snakebite victims is therefore a matter of high priority.

Several methods have been used to reduce acute adverse reactions to antivenom. A small test dose of antivenom to detect patients who may develop acute adverse reactions to the antivenom has no predictive value, can itself give rise to anaphylaxis, and is no longer recommended [Malasit et al., 1986]. Prophylactic use of hydrocortisone and antihistamines before infusion with antivenom is also practised widely, although the theoretical basis for their use is unclear. Antihistamines counter only the effects of histamine after its release and do not prevent further release, and one small randomised controlled trial demonstrated no benefit from the routine use of antihistamines [Fan et al., 1999]. Hydrocortisone takes time to act and may be ineffective as a prophylactic against acute adverse reactions that can develop almost immediately after antivenom treatment, which is very often administered urgently to snakebite victims. A recent study suggests that intravenous hydrocortisone is ineffective in preventing acute adverse reactions to antivenom, but if given together with intravenous chlorpheniramine (an antihistamine) it may reduce these reactions [Gawarammana et al., 2004]. However, this trial recruited only 52 patients and was not designed to study the efficacy of chlorpheniramine alone, making clear interpretation of the results and recommendations on pretreatment with steroids and antihistamines to prevent acute reactions to antivenom difficult.

In one study of 105 patients low-dose adrenaline given subcutaneously immediately before administration of antivenom to snakebite victims significantly reduced the incidence of acute adverse reactions to the serum [Premawardena et al., 1999]. However, the authors were unable to enrol sufficient participants to adequately establish safety, a major concern regarding the use of adrenaline in a prophylactic role [Khanna & Hawkins, 1999], particularly the risk of intra-cerebral haemorrhage [Dassanayake et al., 2002; Horowitz et al. 1996]. Therefore, further studies on the safety of this treatment are required before it can be recommended routinely.

For the present, the only available alternative to prevention is the early detection of adverse reactions to antivenom and the ready availability of drugs such as adrenaline for their prompt treatment. Therefore, the need for a properly controlled trial to study the safety and efficacy of all possible pretreatment medications to prevent acute adverse reactions to antivenom is clear.

# TRIAL DESIGN

**Objective**

To determine whether low-dose adrenaline, promethazine, and hydrocortisone, alone and in different combinations, are significantly better than placebo in the prevention of acute adverse reactions to antivenom in snakebite victims. The principle outcomes will be the development of acute reactions to antivenom. This study also aims to assess the safety of pretreatment medication used before the administration of antivenom.

**Design**

This is a multicentre, double-blind, randomised, placebo-controlled, factorial design clinical trial, to be conducted in two State hospitals in Sri Lanka. The participating hospitals are: General Hospital, Kurunegala and General Hospital, Polonnaruwa, and Base Hospital, Embilipitiya.

**Sample size and predicted events**

We estimate that acute adverse reactions occur in about 40% of patients who receive antivenom. Therefore, proportional reductions of 25-40% of acute adverse reactions would correspond to substantial absolute benefits (see Table). For an anticipated 25% reduction in adverse reactions from the current reaction rate by any one treatment with 80% power at p<0.01 using the proposed design, a sample size of 1000 would be needed for this study.

During the trial it is intended that blinded event rates (i.e. active and placebo groups combined) will be monitored and, if they are substantially lower than anticipated, the TSC will have the option of increasing the sample size.

**Table: Statistical power to detect 25-40% proportional reductions in acute adverse reactions to antivenom among 1000 randomised patients (based on 40% control group event rate)**

| **Proportional**  **reduction** | **Control group**  **500** | **Treatment group**  **500** | **Power for a type I error <0.01** | **Power for a type I error <0.05** | **Events avoided per 1000 treated** |
| --- | --- | --- | --- | --- | --- |
| 40% | 200 | 120 | >95% | >99% | 80 |
| 30% | 200 | 140 | 95% | >95% | 60 |
| 25% | 200 | 150 | 80% | >90% | 50 |

**TRIAL PROCEDURES**

**Eligibility**

To be eligible for entry into the study patients must satisfy **all** the inclusion criteria and **none** of the exclusion criteria.

# Inclusion Criteria

- Above 12 years of age
- Patients admitted to hospital after snakebite in whom antivenom is indicated
- Patients who give informed consent

**Exclusion Criteria**

- Patients who are pregnant or nursing
- Patients who are currently taking beta- or alpha-adrenoceptor antagonists, or tricyclic antidepressants
- Patients in whom adrenaline may be contraindicated (this may include patients with the following: history of ischaemic heart disease, stroke, uncontrolled hypertension, and tachyarrhythmias)

# Information and Consent

The conduct of the study will be in accordance with the Medical Research Council (MRC), UK, Guidelines for Good Clinical Practice in Clinical Trials [MRC, 1998]. Before inclusion of patients in the trial, they will be given full verbal and written information on the nature, objective, significance, expected benefits, and possible adverse effects of the treatment. Full explanation will be given about the manner of treatment allocation, and they will be told that some will not receive active pre-treatment (placebo group). When it is not possible to obtain informed consent from the patient, it will be obtained from a parent, spouse, or guardian. Patients will be told that they are free to withdraw from the trial at any time if they wish to do so, without any prejudicial effects on their subsequent management.

# Assessment of patients

All patients presenting to hospital after snakebite will be registered. However, only those who satisfy inclusion criteria will be included in the trial. Assessment of eligibility criteria for recruitment to the trial will be done by Research Assistants (see below) in consultation with attending clinicians in the hospital wards. Clinical assessment of patients and clinical decision-making during the trial will be done by attending clinicians at participating trial centres. They and other clinical ward staff involved with patient management will be blinded to the interventions in the trial. In addition they will follow a standard management protocol to further reduce possible bias.

Patients who are eligible to enter the trial will undergo the following before any trial medication is given (baseline):

- Clinical assessment, including full neurological evaluation
- Assessment of blood clotting
- Electrocardiography
- Any other investigation requested by the attending doctor

# Randomization

Registration of subjects, randomisation, supervision of intervention, and maintenance of trial records will be performed by medically qualified Research Assistants (RA), dedicated to the trial, who will be available throughout the 24 hours at each participating trial centre.

Randomisation to one of the treatment arms will be done after inclusion in the trial, and where necessary after resuscitation has been given. Randomisation will be based on a computer generated random table, which will be preplanned at the trial coordinating centre in the Faculty of Medicine, University of Kelaniya. There will be three different blocks of random numbers for each participating trial centre. All trial medications will be prepared at the trial coordinating centre and will be packaged in numbered sealed envelopes which will be stored in refrigerators. Syringes containing adrenaline and adrenaline placebo will be clearly marked to ensure that they are not administered intravenously. All envelopes will have a unique, centre-specific, ID number, and will be kept at the trial centres with the RAs, who will have no role in the treatment or assessment of the patients. Clinicians in the trial centres will not have access to these envelopes, and therefore have no role in the randomisation process. If the patient is eligible and informed consent has been obtained, the patient will be randomised and given a unique sequential centre-specific identifier, which will be entered in the clinical record form (CRF). This number will correspond to the number on the sealed envelope.

Envelopes will be opened **after** eligibility criteria have been confirmed and randomisation is done. The RAs will open the sealed envelope bearing the corresponding number to the randomisation number and handover the pre-prepared trial medication to the clinical ward staff who will administer them to patients. Both RAs and the clinical staff will be blind to the intervention.

# Treatment plan

Consecutive patients who are admitted after snakebite and show evidence of systemic envenomation or severe local envenomation should be given antivenom. This decision will be taken by the clinicians at trial centres. Trial medication, which will be administered to all randomised patients before antivenom therapy include:

- Adrenaline (0.25 ml of 1:1000 adrenaline) subcutaneously given to an arm (over the deltoid) which has not been envenomed
- Promethazine 25 mg intravenously
- Hydrocortisone 200 mg intravenously
- 0.9% NaCl (placebo)

These will be given immediately before antivenom infusion is started (as pretreatment). No test dose of antivenom will be used. Eligible patients will be randomised in a factorial (2 x 2 x 2) blinded design using a treble-dummy technique (Figure) between:

- Adrenaline 0.25 ml sc
- Promethazine 25 mg iv
- Hydrocortisone 200 mg iv
- Adrenaline 0.25 ml sc & hydrocortisone 200 mg iv
- Adrenaline 0.25 ml sc & promethazine 25 mg iv
- Promethazine 25 mg iv & hydrocortisone 200 mg iv
- Adrenaline 0.25 ml sc & promethazine 25 mg iv & hydrocortisone 200 mg iv
- No active pretreatment (placebo sc and iv)

The efficiency of a 2 x 2 x 2 factorial design will allow separate assessment of all study questions without any material effect on non-drug cost or sample size requirements. The primary analyses will involve three-way comparisons of:

- All those allocated adrenaline versus all those who were not allocated adrenaline, irrespective of the allocation of other drugs
- All those allocated promethazine versus all those who were not allocated promethazine, irrespective of the allocation of other drugs
- All those allocated hydrocortisone versus all those who were not allocated hydrocortisone, irrespective of the allocation of other drugs

Figure: Factorial design of trial

| Adrenaline 0.25 ml sc  +  Placebo 1ml iv  +  Placebo 1 ml iv | Adrenaline 0.25 ml sc  +  Hydrocort 200 mg iv  +  Placebo 1 ml iv | Adrenaline 0.25 ml sc  +  Hydrocort 200 mg iv  + Promethazine 25 mg iv | Adrenaline 0.25 ml sc  +  Promethazine 25 mg iv  +  Placebo 1 ml iv |
| --- | --- | --- | --- |
| Placebo 0.25 ml sc  +  Placebo 1 ml iv  +  Placebo 1 ml iv | Hydrocort 200 mg iv  +  Placebo 0.25 ml sc  +  Placebo 1 ml iv | Hydrocort 200 mg iv  +  Promethazine 25 mg iv  +  Placebo 0.25 ml sc | Promethazine 25 mg iv  +  Placebo 1 ml iv  +  Placebo 0.25 ml sc |

All patients included in the trial will be given 10 vials of antivenom dissolved in 500 ml of isotnic saline as an intravenous infusion over one hour. Antivenom infusion will be given immediately after the administration of trial medications. Antivenom treatment may be repeated if deemed necessary by attending clinicians according to clinical judgment. However, patients will not be given further / repeat doses of trial medication, even if antivenom is repeated.

## Adverse effects of antivenom and trial medication

Patients will be monitored for development of acute adverse reactions to antivenom and any adverse reactions to the test drugs as follows:

- Pulse rate, BP, temperature, auscultatory signs in the lungs, and allergic reactions (see below) will be monitored every 5 minutes for 20 minutes, then every 15 minutes for 120 minutes, and thereafter 4 hourly for 48 hours
- ECG (within the 30 minutes following end of infusion and whenever clinically indicated)
- Neurological evaluation at the end of antivenom infusion and 3 hours after the infusion, and whenever clinically indicated
- Peak flow measurement before and at the end of antivenom infusion, and whenever clinically indicated
- Observation for any adverse reactions until discharge from hospital

Patients who receive antivenom will be kept in hospital for at least 96 hours after the infusion. If a reaction develops during infusion or if the patient develops arrhythmias, ischaemic changes on the ECG, a measurable rise in BP (systolic > 30 mmHg or diastolic > 20 mmHg), a measurable drop in BP (systolic > 20 mmHg or diastolic > 10 mmHg), or anaphylaxis after the test drug and antivenom, he / she will be given appropriate treatment as decided by the attending clinicians (rescue medication). Antivenom reactions will be treated by stopping the antivenom infusion temporarily, administering either 0.25 ml of 1:1000 adrenaline (mild reactions) or 0.5 ml of 1:1000 adrenaline (moderate and severe reactions) intramuscularly and whatever other medications are deemed necessary by the attending clinicians. These, if given, will be recorded in the patient CRF.

# ASSESSMENT OF OUTCOME

**Primary outcomes:**

The primary analysis will compare the frequency of **severe reactions** to antivenom in the first 48 hours, among all those allocated each separate treatment versus all those allocated placebo for that treatment, i.e.:

1. all those allocated adrenaline versus all those allocated no adrenaline
2. all those allocated promethazine versus all those allocated no promethazine
3. all those allocated hydrocortisone versus all those allocated no hydrocortisone

**Secondary outcomes:**

Comparisons will be made of the effects of each of the study treatment allocations on rates of severe reactions at first 1 or 6 hrs, rates of any adverse reactions (mild, moderate, and severe) in the first 48 hrs, and acute adverse reactions to study treatments separately.

Classification of adverse reactions:Tertiary (Brown, 2004):

- Mild – fever, rigors, pruritus, urticaria, facial oedema
- Moderate – bronchospasm, stridor, abdominal pain, nausea, vomiting
- Severe – hypotension SBP below 80 mmHg, cyanosis, confusion, altered or loss of consciousness

Specified adverse reactions to study treatments

- Development of arrhythmias, ischaemic changes on the ECG after the trial drug and antivenom
- A significant rise in blood pressure (systolic > 30 mmHg or diastolic) after the trial drug and antivenom
- Intracerebral haemorrhage

**Adverse events**

All patients will be closely observed for any adverse events (rise in blood pressure, ischaemic changes in the ECG, etc) after treatment with antivenom or trial medications. Both adrenaline and promethazine are widely used in clinical practice, but all serious or unexpected adverse events will be recorded in the patient CRF and reported to the consultant physicians (in charge of the medical units in trial centres) and the trial statistician. Serious adverse events are those which are fatal, life-threatening or disabling, or require prolongation of hospitalisation. These reports will be reviewed immediately, blind to treatment allocation by the TSC, and any further information required will be sought urgently. Confirmed reports will then be promptly forwarded ‘unblinded’ to the Chairman of the Data Monitoring Committee by the trial statistician. It is not required to report in this way non-serious adverse effects of test medications.

If any patient develops serious adverse reactions these will be recorded, and he / she will be given appropriate treatment as indicated above.

**DATA ANALYSIS**

# Methods of analysis

The fundamental assessments of efficacy will involve comparisons of the proportions of patients experiencing an event among all the randomised patients in their originally allocated treatment group, irrespective of what treatment they were actually given (i.e. “intention-to-treat” analyses). Two-sided P values will be used. No allowance will be made for multiple hypothesis testing in the primary comparison of each of the three study treatments in this 2 x 2 x 2 factorial study. For secondary and, particularly, for tertiary comparisons, allowance will be made for multiple hypothesis testing, taking into account the nature of the events (including timing, duration, and severity) and evidence from other studies.

The independent effects and effects of interactions of the three interventions will be assessed by using Cox regression. Cox regression will be used with a constant time to event for all subjects as we are only interested in reaction rate and not in the time to reaction.

# TRIAL SUPERVISION

# Trial steering committee

The Trial Steering Committee (TSC Chairman: Prof Janaka de Silva) from the Faculty of Medicine, University of Kelaniya, will have overall responsibility for the trial, and will coordinate and supervise the study. They will not be directly involved with the conduct of the study or patient management / assessment. Supervisory visits to trial centres will be made by members of the TSC at regular intervals (usually once in 3-4 weeks) to monitor trial progress and ensure adherence to the trial protocol by the investigators in the trial centres. Patient CRFs will be evaluated during each visit and those that are complete will be retained for subsequent analysis.

The main function of the TSC is to maintain overall integrity of the trial. The Sri Lankan members of the TSC will meet at least once a month until the end of the trial. The UK members will be kept informed of progress after each meeting.

**DATA MONITORING**

**Interim analyses and the role of the Data Monitoring Committee**

The Trial Steering Committee will appoint an independent Data Monitoring Committee consisting of a Chairman, statistician and a clinician.

During the study, the Data Safety Monitoring Committee will be informed of any serious adverse events. The trial statistician will prepare unblinded data for examination by the Data Monitoring Committee when information from the first 200 patients becomes available and again when 400 patients have been analysed.  In the light of these analyses and the results of any other new relevant information, the Data Monitoring Committee will advise the Steering Committee if, in their view, the randomised comparisons have provided both; (i) "proof beyond reasonable doubt" that any of the study treatments is clearly indicated or clearly contraindicated in terms of a net difference in severe adverse reactions, and (ii) evidence that might be reasonably expected to influence the management of many clinicians.  In general a difference of at least 3 standard deviations (i.e. P<0.001) in an interim analysis of a major endpoint would be needed to justify halting, or modifying, the study prematurely, especially if the comparison were based on relatively few events (e.g. under 100). If this criterion is adopted, it has the
practical advantage that the exact number of interim analyses would be of little importance.

Unless this happens, the Steering Committee, investigators, and the coordinating centre staff (except those who supply the confidential analyses) will remain ignorant of the interim results on adverse reactions until the study is terminated. During the study, investigators and all others associated with the study may write to the chairman of the Data Monitoring Committee, drawing attention to any worries they may have about the possibility of particular adverse effects, or about any other matters that may be relevant.

The trial statistician will monitor recruitment to the trial, protocol adherence, and serious adverse events. The TSC will consider reports made by the trial statistician and any other relevant studies published during the timeframe of this trial. Recommendations made by the TSC will be passed by its Chairman to the attending clinicians in the three trial centres.

**REFERENCES**

1. Ariaratnam CA, Sjostrom L, Raziek Z, et al. An open, randomised controlled trial of two antivenoms for the treatment of envenoming by Sri Lankan Russell’s viper (Daboia russelli russelli). Trans R Soc Trop Med Hyg 2001; 95: 74-80
2. Brown SGA. Clinical features and severity grading of anaphylaxis. J Allergy Clin Immunol 2004; 114: 371-6
3. Dassanayake AS, Karunanayake P, Kasturiratne KT, et al. Safety of subcutaneous adrenaline as prophylaxis against acute adverse reactions to anti-venom serum in snakebite. CMJ; 2002; 47: 48-9
4. De Silva A, Ranasinghe L. Epidemiology of snakebite in Sri Lanka. CMJ 1983; 28: 144-54
5. Fan HW, Marcopito LF, Cardoso JL, Franca FO, Malaque CM, Ferrari RA et al. Sequential randomised and double blind trial of promethazine prophylaxis against early anaphylactic reactions to antivenom for Bothrops snake bites. BMJ 1999; 318:1451-2
6. Gawarammana IB, Abeysinghe S, Kularatne M, et al. Parallel infusion of hydrocortisone ± chlorpheniramine bolus injection to prevent acute adverse reactions to antivenom for snakebites: a randomised, double-blind, placebo-controlled study. MJA 2004; 180: 20-3
7. Horowitz BZ, Jadallah S, Derlet RW. Fatal intracranial bleeding associated with peripheral use of epinephrine. Ann Emerg Med 1996; 28: 725-7
8. Karunaratne KE de S, Anandadas JA. The use of anti-venom in snakebite poisoning. CMJ 1973; 1: 37-43
9. Khanna R, Hawkins WJ. A plea for caution in the use of adrenaline. BMJ (electronic version); bmj.com, 19 May, 1999
10. Kularatne SAM. Reactions to snake venom antisera: study of pattern, severity and management at general Hospital Anuradhapura. Sri Lanka J Med 2000; 9: 8-13
11. Malasit P, Warrell DA, Chanthavanich P, et al. Prediction, prevention and mechanism of early (anaphylactic) antivenom reactions in victims of snakebite. BMJ 1986; 292: 17-20
12. Medical Research Council Guidelines for Good Clinical Practice in Clinical Trials. Medical Research Council 1998. (http://www.mrc.ac.uk/pdf-ctg.pdf)
13. Premawardena AP, de Silva CE, Fonseka MMD, et al. Low dose subcutaneous adrenaline to prevent acute adverse reactions to antivenom serum in snake bite: a randomised placebo-controlled trial. BMJ 1999; 318: 730-3
14. Theakston RDG, Philip RE, Warrell DA, et al. Envenoming by the common krait (*Bungarus caeruleus*) and Sri Lankan cobra (*Naja naja naja*): efficacy and complications of therapy with Haffkine antivenom. Trans R Soc Trop Med Hyg 1990; 84: 301-8
